# 56 Friction Burns: Defining the Rub of Road Rash

**DOI:** 10.1093/jbcr/irae036.048

**Published:** 2024-04-17

**Authors:** Louis Perkins, Sadie Munter, William A Johnston, Jeanne Lee, Laura Haines, Jay Doucet, Jarrett E Santorelli

**Affiliations:** University of California San Diego, San Diego, California; University of California San Diego, San Diego, CA; University of California San Diego, San Diego, California; University of California San Diego, San Diego, CA; University of California San Diego, San Diego, California; University of California San Diego, San Diego, CA; University of California San Diego, San Diego, California; University of California San Diego, San Diego, CA; University of California San Diego, San Diego, California; University of California San Diego, San Diego, CA; University of California San Diego, San Diego, California; University of California San Diego, San Diego, CA; University of California San Diego, San Diego, California; University of California San Diego, San Diego, CA

## Abstract

**Introduction:**

There is a paucity of literature on friction burns. The involvement of burn surgery teams and the need for surgical intervention in these cases remain relatively unexplored. This study aims to characterize friction burns resulting from motorcycle accidents, evaluate the role of specialized burn surgery teams, and assess the need for operative intervention.

**Methods:**

The trauma registry of a Level 1 Trauma Center was queried for all admissions after motorcycle accidents between January 2018 and December 2022. Patients were included if there was an external cause of injury code for an abrasion. Abstracted data included demographics, location of abrasion, injury severity score, and presence of protective clothing. Further chart review was conducted to confirm a road rash injury, identify burn consultation, need for operating room (OR) procedure with burn service, need for skin grafting, days of burn nurse care, wound care recommendations, and burn outpatient follow-up. Potentially unnecessary burn consults were defined as consults which required no burn nursing care and where outpatient follow-up was not planned. Multivariate logistic regression was used to explore trends in burn consultation.

**Results:**

Among the 810 patients meeting inclusion criteria, the cohort was 91.5% male, 45.6% Hispanic, with median age of 33 (IQR 26-44), and median ISS 8 (IQR 5-14). 32.7% of patients were wearing protective clothing at the time of injury. The extremities were most affected by friction burn followed by the abdomen, thorax, and face. Burn surgery was consulted in 6.9% (n=56) of cases which had a median TBSA of 3.75% (IQR 2.0-5.2%); 21.4% (n=12) of these patients underwent excision in the OR, 16.1% (n=9) required autografting, and 60.7% were referred for burn outpatient follow-up. Consults to burn were more likely in female (OR 3.67, p=0.006) and African American patients (OR 3.73, p=0.023) and those with involvement of the upper extremity, abdomen, or thorax. The median days of burn nursing care was one day (IQR 0 – 5.3 days) and 39.4% of consults made by non-burn trained trauma providers were suspected to be unnecessary.

**Conclusions:**

We have provided a better understanding of the characteristics and treatment of friction burns, which have previously been under studied in the burn literature. Local wound care is sufficient for a vast majority of these injuries and the need for specialized burn care and operative intervention is rare.

**Applicability of Research to Practice:**

Applicable; this study characterizes the nature of friction burns after motorcycle trauma and the role of burn surgery teams in evaluation and treatment.

